# Evaluation of ^177^Lu-Labeled Lipiodol as a Targeted Radionuclide Therapy for Hepatocellular Carcinoma in a Preclinical Xenograft Model

**DOI:** 10.1007/s11307-025-02016-1

**Published:** 2025-06-04

**Authors:** Yumiko Kono, Keita Utsunomiya, Takahiro Shiraishi, Naoki Kan, Ichiro Shiojima, Kaoru Maruyama, Noboru Tanigawa

**Affiliations:** 1 Department of Radiology, Kansai Medical University, 2-5-1 Shin-Machi, Hirakata City, Osaka, 573-1010 Japan; 2 Radioisotope Research Center, Kansai Medical University, 2-5-1 Shin-Machi, Hirakata City, Osaka, 573-1010 Japan; 3 Department of Radiology, Sumitomo Hospital, 5-3-20 Nakanoshima, Kitaku, Osaka City, Osaka, 530-0005 Japan

**Keywords:** Lutetium-177, Lipiodol, Hepatocellular carcinoma, Radionuclide therapy, Biodistribution, Tumor growth suppression, SPECT imaging, Theranostics

## Abstract

**Background:**

Lutetium-177 (^177^Lu) is a promising radionuclide for targeted cancer therapy due to its favorable theranostic properties. Transarterial lipiodol embolization is widely used for hepatocellular carcinoma (HCC), but the potential of ^177^Lu emulsified into lipiodol (^177^Lu-lipiodol) remains underexplored. This study aimed to evaluate the partition coefficient, biodistribution, and antitumor efficacy of ^177^Lu-lipiodol in a preclinical xenograft model.

**Methods:**

After synthesizing ^177^Lu-oxine from ^177^Lu-chloride, the product was emulsified in lipiodol. Its radiochemical purity and partition coefficient were measured. F344 NJcl rnu/nu rats (n = 5) bearing bilateral thigh tumors (HC-4 cells) were randomized to receive ^177^Lu-lipiodol (2.8 MBq in 50 μL) or non-labeled lipiodol (50 μL) via surgical exposure and direct puncture of the right femoral artery. SPECT/CT images were acquired over 14 days, and biodistribution was confirmed by gamma counting at day 28. Tumor volumes and body weights were monitored to assess treatment response and toxicity.

**Results:**

The ^177^Lu-lipiodol emulsion was obtained with a high radiochemical purity (> 99%). SPECT/CT showed high tumor accumulation (34.0% ± 4.4% immediately post-injection) that persisted up to day 28 (7.3% ± 1.2% of injected dose). Tumor growth was significantly suppressed with a treated-to-untreated volume ratio of 0.45 at day 14 (p = 0.017) and 0.59 at day 21 (p = 0.001). While off-target uptake was limited, moderate splenic accumulation (26.6% ± 17.5% ID) was noted. No marked body weight changes or gross organ abnormalities were observed.

**Conclusion:**

^177^Lu-lipiodol for HCC therapy demonstrated effective tumor targeting and growth suppression of HCC in a preclinical xenograft model.

**Supplementary Information:**

The online version contains supplementary material available at 10.1007/s11307-025-02016-1.

## Background

Lutetium-177 (^177^Lu) has emerged as a promising radioisotope for targeted cancer therapy owing to its favorable beta energy, gamma co-emission, and theranostic potential [1]. Various ^177^Lu-based agents, such as ^177^Lu-PSMA ligands [2, 3, 4] or ^177^Lu-DOTATATE [5, 6], have shown clinical efficacy in prostate cancer and neuroendocrine tumors, respectively. Meanwhile, lipiodol, a poppy-seed oil–based radiopaque agent, has long been employed in transarterial embolization for treating hepatocellular carcinoma (HCC) [7, 8. Due to HCC’s hypervascularization and sluggish venous outflow [9, 10], lipiodol exhibits selective retention in tumor nodules, making it an attractive carrier for therapeutic radionuclides. However, iodine‐131-lipiodol has encountered practical challenges related to its high-energy gamma emissions [11]. Rhenium-188-labeled lipiodol has also been explored [12, 13], but its relatively short half-life (~ 17 h) has limited sustained tumor irradiation. On the other hand, ^177^Lu-lipiodol, combined with SPECT-based imaging, offers a potentially superior half-life (~ 6.7 days) for prolonged therapeutic effect. Nevertheless, only limited preclinical data exist regarding its therapeutic efficacy and biodistribution [14]. This study aimed to evaluate the feasibility, biodistribution, tumor retention, and antitumor effects of ^177^Lu-lipiodol in an HCC rat xenograft model. We also conducted a basic toxicity assessment via body weight monitoring and gross organ inspection.

## Materials and Methods

### Cell Line and Chemicals

Human hepatocarcinoma cells (HC-4), obtained from the Cell Resource Center for Biomedical Research, Cell Bank (Tohoku University, Japan) were used in this study. A mycoplasma contamination test was performed before transplantation cells into the animals, which was confirmed to be negative. Cells were maintained in Eagle Minimum Essential Medium (Eagle MEM; FUJIFILM Wako Pure Chemical Corporation, Japan) supplemented with 10% fetal bovine serum (FBS; Gibco, Thermo Fisher Scientific, Waltham, MA, USA). ^177^Lu-chloride (^177^LuCl_3_; PDRadiopharma, Japan) and Lipiodol (Guerbet, France) are analytical grade and were used without purification.

### *In Vitro* Cell Irradiation and Replication Assay

To confirm HCC radiosensitivity, we performed a gamma-irradiation replication assay, following our previously reported protocol [15]. The HC-4 cells were irradiated with gamma rays in a single-cell suspension using a gamma cell 40 Exactor (Nordion International; dose rate 0.60 Gy/min) (0, 3, 6, 9, or 12 Gy). Five groups categorized according to the radiation dose were prepared: 0 Gy (control group), 3 Gy (3 Gy group), 6 Gy (6 Gy group), 9 Gy (9 Gy group), and 12 Gy (12 Gy group). At days 3, 5, 7, and 14, cells were harvested, stained with trypan blue, and counted. The replication rate (RR) was calculated according to Eq. (1):

1 $$\text{RR}=\frac{{\text{N}}_{\text{irradiated}}}{{\text{N}}_{\text{control}}}$$

N_irradiated​_ is the number of viable cells in the irradiated group, and N_control_ is that in the non-irradiated control group.

### Synthesis of ^177^Lu-Oxine and Emulsification into Lipiodol

^177^Lu-oxine was prepared according to a published procedure [16]. Briefly, a measured activity (60 MBq, 2.49 µL) of ^177^LuCl_3_ was combined with 0.5 M oxine in ethanol and 20 mM ammonium acetate buffer (pH 6.5), heated at 50 °C for 30 min, then cooled and extracted with dichloromethane. After drying under nitrogen, the ^177^Lu-oxine residue was obtained. The radiochemical purity (≥ 99%) was confirmed by performing thin-layer chromatography (TEC-CONTROL Chromatography Strips (Black), Mirion Technologies (Capintec), Inc., Florham Park, New Jersey, USA) using methanol as the solvent. Dried ^177^Lu-oxine (37 MBq) was mixed with 0.6 mL lipiodol (50 °C) for 1 h in a 50 °C water bath to obtain ^177^Lu-lipiodol. Afterward, 0.6 mL saline was added, and the biphasic mixture was vigorously vortexed, then allowed to stand for 5 min. The lipiodol phase (containing ^177^Lu-oxine) was separated and measured with a curiemeter. To assess lipophilicity, a fixed amount of 177Lu-lipiodol was mixed with saline, centrifuged,

and the radioactivity in each phase was measured. The partition coefficient (log P) was calculated according to Eq. (2):

2 $${\text{log}}_{10}\left(\frac{\mathrm{activity\; in\; oil\; phase}}{\mathrm{activity\; in\; aqueous\; phase}}\right)$$

### Tumor Embolization Study *In Vivo*

All animal experiments were approved by the Institutional Animal Care and Use Committee of Kansai Medical University (Approval Number: 24–057).

### Tumor Embolization Study *In Vivo*

Five male F344 NJcl rnu/nu rats (6–8 weeks old) were subcutaneously inoculated with 5.0 × 10^6^ HC-4 cells in each thigh. Tumors were allowed to grow for five weeks, reaching approximately 0.3 cm^3^ in volume. Three rats (RI-group) received ^177^Lu-lipiodol (2.8 MBq in 50 μL), and two rats (C-group) received non-labeled lipiodol (50 μL). Under isoflurane anesthesia, a small groin incision was made to expose the right femoral artery, which was directly punctured (29G needle), and the lipiodol emulsion was slowly injected under fluoroscopic guidance. The artery was ligated post-injection, and the incision was sutured.

### SPECT/CT Imaging

All imaging was performed on an Inveon® multimodality system (Siemens Medical Solutions, USA). CT was acquired with a transaxial field of view (FOV) of 106.73 mm in a step-and-shoot mode, and SPECT was performed with a tungsten collimator (3-RWB-1.8). Reconstruction was performed using the MAP3D algorithm (PSF correction, 16 iterations, 6 subsets). Immediately after ^177^Lu-lipiodol administration and on days 3, 7, and 14, we evaluated the accumulation rate (AR) in the tumor and lungs relative to the whole-body ROI (ROI_WB_)*.* Equations (3) and (4) define AR in the tumor and lungs, respectively:

3 $${\text{AR}}_{\text{tumor}}= \frac{{\text{ROI}}_{\text{tumor}}}{{\text{ROI}}_{\text{WB}}}\times 100 \%$$

4 $${\text{AR}}_{\text{lung}}= \frac{{\text{ROI}}_{\text{lung}}}{{\text{ROI}}_{\text{WB}}}\times 100 \%$$

ROI_tumor_ was defined as a 10 cm^3^ spheroid region centered on the tumor in the lower leg of the treatment side, ROI_lung_ was defined as a 90 cm^3^ long rectangular region centered on the lung region, and ROI_WB_ as an 1100 cm^3^ long rectangular region that contained the entire rat. The accumulation rate in the tumor and lung of the treatment group was evaluated over time.

Tumor volumes were measured on days 3, 7, 14, 21, and 28 via ultrasound. Rats were euthanized on day 28. Tumors and major organs (e.g., liver, spleen, lungs, kidneys, bone, blood) were harvested, weighed, and their radioactivity was measured using a gamma counter (WIZARDTM 3′ 1480, PerkinElmer Life Sciences) to yield the percentage of injected dose per gram of tissue (% ID/g). The ID/g was calculated using the dilution standard. Body weight was monitored as an indicator of systemic toxicity. Gross inspection of organs was performed to identify morphological abnormalities. Hematological parameters were not measured in this pilot study.

### Evaluation of Antitumor Effect and Side Effects

To observe the treatment effect, the tumor volume of both thighs was measured by ultrasound on days 3, 7, 14, 21, and 28 after the treatment, and growth rate was obtained and growth rate ratio (T/U ratio) between the treated and untreated sides were calculated Eqs. (5) and (6). At the same time point, body weight was also measured, and the weight change rate was calculated according to Eq. (7) to assess transient toxicity.

5 $$\text{Growth rate}= \frac{\text{Tumor Volume at each time point}}{\text{Baseline tumor volume}\,(\text{day}0)}$$

6 $$\text{T}/\text{U ratio }=\frac{\text{tumor growth rate}}{\text{Untreated tumor growth rate}}$$

7 $$\text{Weight change rate }=\frac{\text{Body weight at each time point}}{\text{Baseline body weight }(\text{day}0)}$$

On day 28, post-treatment dissection of all organs was visually inspected.

### Statistical Analysis

Statistical analysis of the results was performed using repeated measures ANOVA, and Tukey’s post-hoc test was subsequently applied. Group differences between the RI-group and C-group at each time point were analyzed using independent sample t-tests, and the p-values were adjusted for multiple comparisons using Bonferroni correction. *In vivo* tumor growth rate was compared between the groups at each time point using the t-test and Bonferroni correction was applied to correct for the time effect. The statistical significance was set at p < 0.05.

## Results

### Replication Study *In Vitro*

**Fig. 1:**
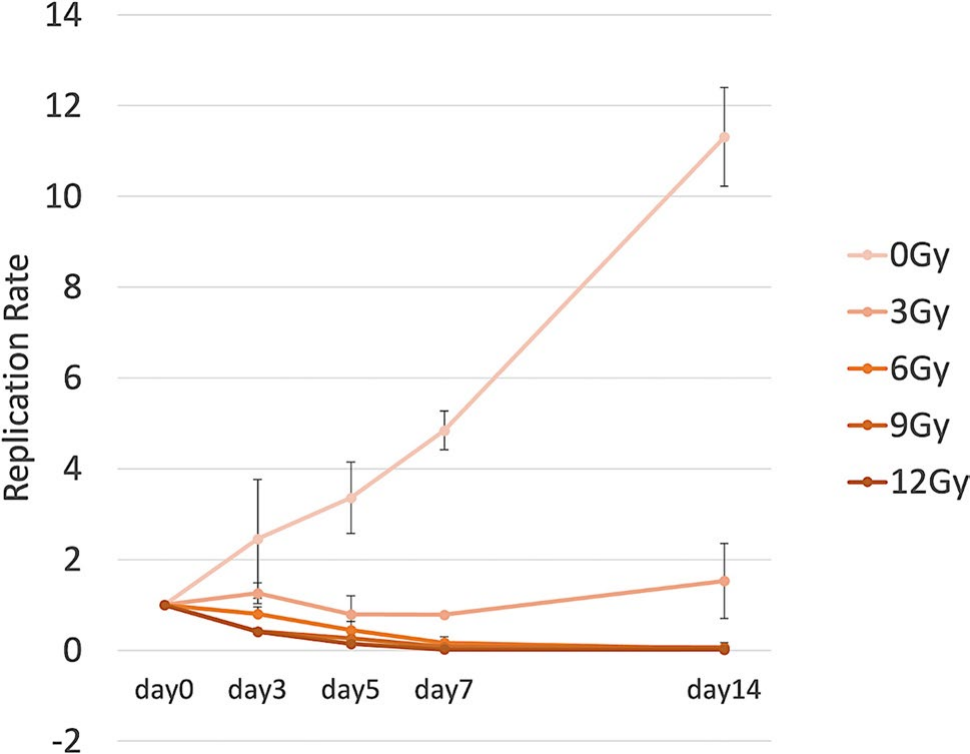
Effect of gamma irradiation on replication rate of HC-4 cells. The replication rate was measured over 14 days at different radiation doses (0, 3, 6, 9, and 12 Gy)

Using Eq. (1), the replication rate of all irradiated HC-4 groups (3–12 Gy) was significantly lower than that of the non-irradiated control group (p = 0.0024). The dose-dependent effect persisted throughout the 14-day observation period (Fig. 1).

### Preparation and Characterization of 177Lu-Lipiodol

The radiochemical yields of ^177^Lu-oxine after the reaction and before evaporation were 98.0% and 99.8%, respectively. After emulsification with Lipiodol, 99.1% was contained in the oil layer, and log P was approximately 2.04, indicating moderate lipophilicity.

### Biodistribution Study

**Fig. 2:**
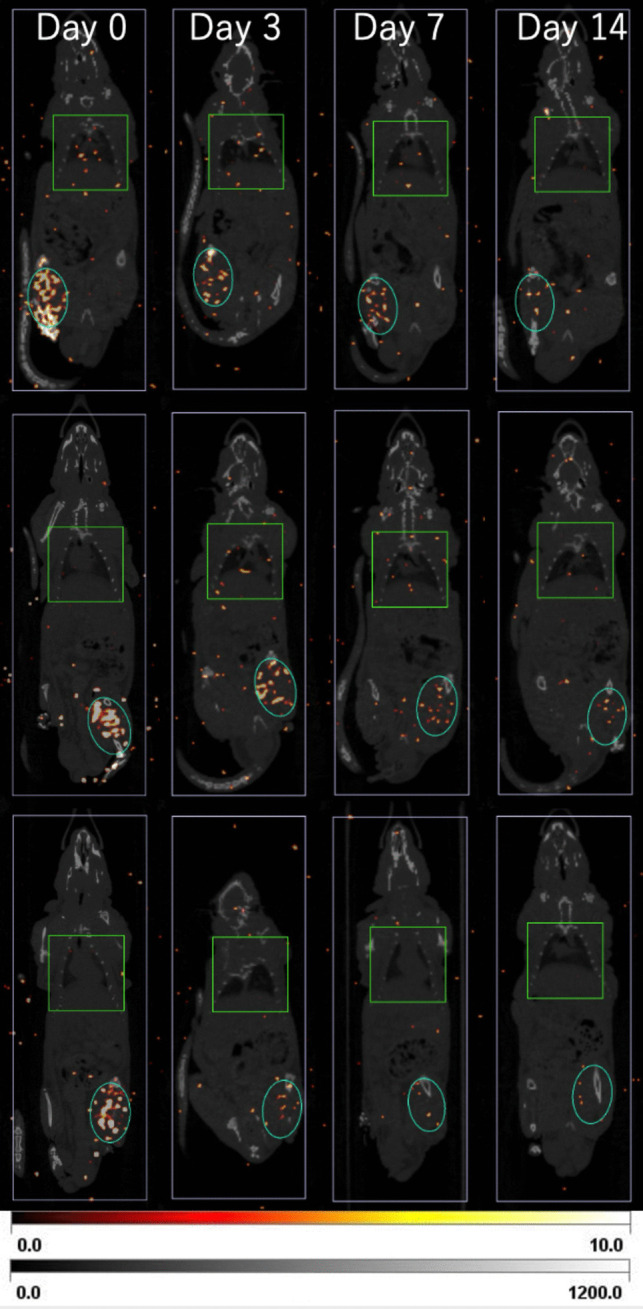
SPECT/CT images showing ^177^Lu-lipiodol biodistribution. The images, featuring ROI_tumor_, ROI_lung_ and ROI_WB_, were acquired on days 0, 3, 7, and 14. High tumor-specific accumulation is evident in the lower leg, while only minimal non-target uptake was observed in other regions, with temporary accumulation in the lungs on day 3

Following embolization therapy, the RI-group showed a high level of accumulation on SPECT/CT which was limited to the lower leg on the treatment side, and this accumulation decreased over time (Fig. 2). Minimal accumulation was observed in off-target areas, except for the lungs, which showed a slight transient uptake, peaking on day 3. This visual evidence supports the tumor selectivity and retention of ^177^Lu-lipiodol. ${\text{AR}}_{\text{tumor}}$(Eq. (3)) was 34.0 ± 4.4% (mean ± SD) immediately after embolization, 17.9 ± 4.4% on day 3, 12.8 ± 5.4% on day 7, and 6.0 ± 2.6% on day 14. The ${\text{AR}}_{\text{lung}}$(Eq. (4)) was 4.1 ± 0.6% immediately after embolization, 8.6 ± 1.7% on day 3, 7.0 ± 1.0% on day 7, and 5.6 ± 1.2% on day 14, showing a slight increase in accumulation that peaked on day 3. ROI data for each rat are shown in Appendix Table 1.

**Table 1:** The tumor growth rate after treatment (unitless)

Time point	RI-group	C-group	p value	Adjusted p-value
day0	1.0 ± 0.00	1.0 ± 0.00		
day3	0.85 ± 0.03	0.89 ± 0.00	0.789	1.000
day7	0.56 ± 0.35	1.06 ± 0.24	0.210	1.000
day14	0.45 ± 0.66	1.39 ± 0.71	0.060	0.360
day21	0.59 ± 2.37	3.94 ± 3.06	0.002	0.012
day28	1.60 ± 2.60	5.28 ± 3.30	0.022	0.133

Data are: Average ± standard deviation

**Fig. 3:**
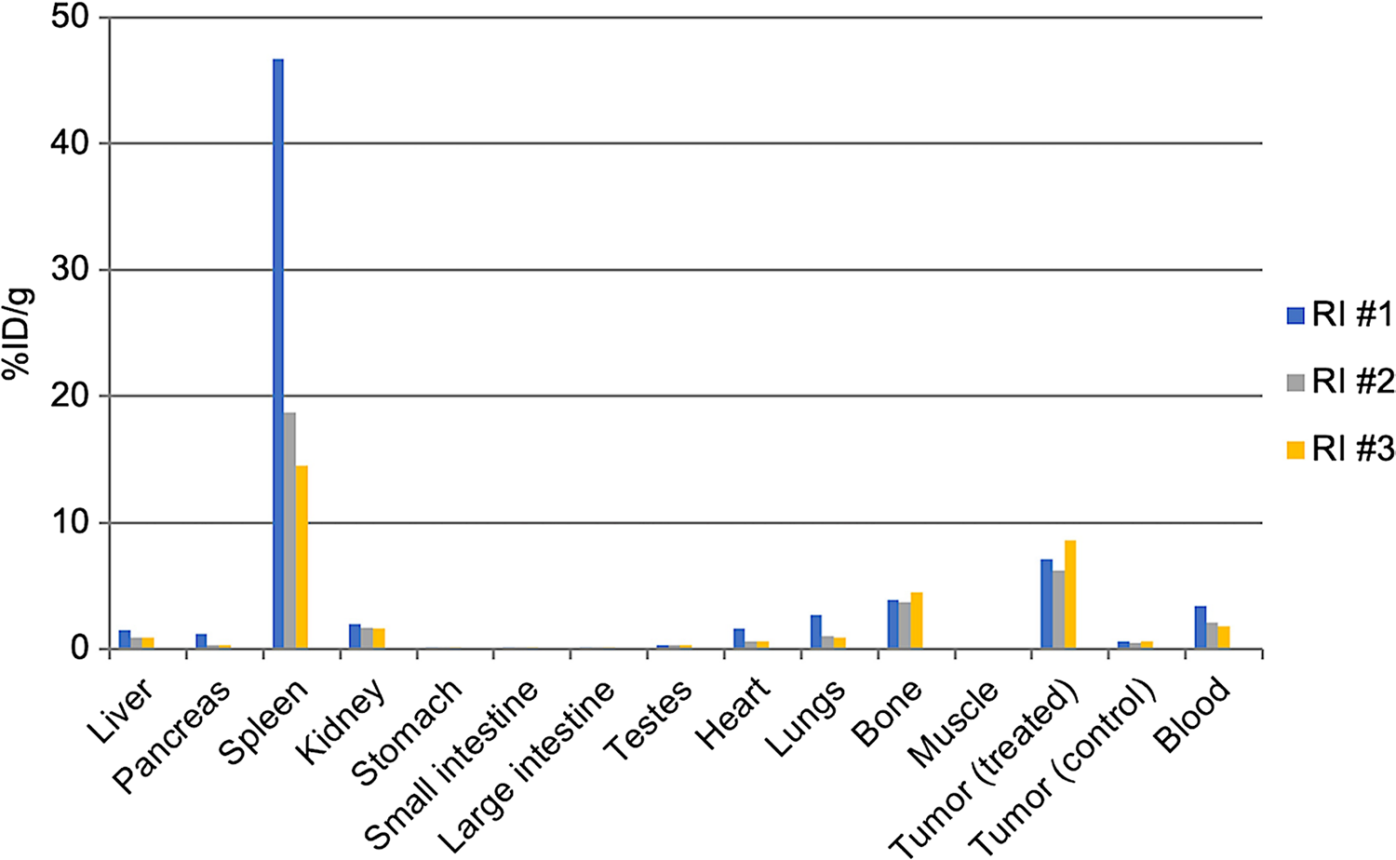
Biodistribution of ^177^Lu-lipiodol on day 28. %ID/g values showed selective retention in tumors and spleens, with minimal accumulation in other tissues, indicating tumor-specific targeting and limited systemic exposure

Organ %ID/g was calculated at the autopsy on day 28 after embolization (Fig. 3). The graph shows the percentage of injected dose per gram of tissue (%ID/g) for treated tumors, control tumors, and other major organs in three individual rats (RI #1, RI #2, and RI #3). Despite a prolonged observation period until day 28, the residual ^177^Lu in the tumor was 7.3 ± 1.2%ID/g. On the other hand, a higher accumulation of 26.6 ± 17.5%ID/g was found in the spleen, followed by residual accumulation of 4.1 ± 0.4%ID/g and 2.4 ± 0.9%ID/g in the bone and blood, respectively. Accumulation of ^177^Lu in the other organs was lower than that in the blood. This highlights the selective retention of ^177^Lu-lipiodol in the target tissues while minimizing systemic exposure.

### Evaluation of Antitumor Effect and Side Effects

The tumor growth rates of the RI-group and C-group after treatment are summarized in Table 1. While the untreated tumors in both groups showed an increase over time, the tumors on the treatment side in each group showed a decrease in tumor accumulation; the average growth rate (Equation (5)) was 0.85 in the RI-group and 0.89 in the C-group on the third day of treatment. After the seventh day of treatment, the tumor on the treatment side in the C-group began to increase in size again, whereas the tumor size in the RI-group continued to decrease until day 21. However, the tumor growth rate in the RI-group exceeded one, and regrowth was observed on day 28. At day 21, the tumor treated with ^177^Lu-lipiodol was significantly smaller than that of the C-group, indicating an antitumor effect (p=0.01).

**Fig. 4:**
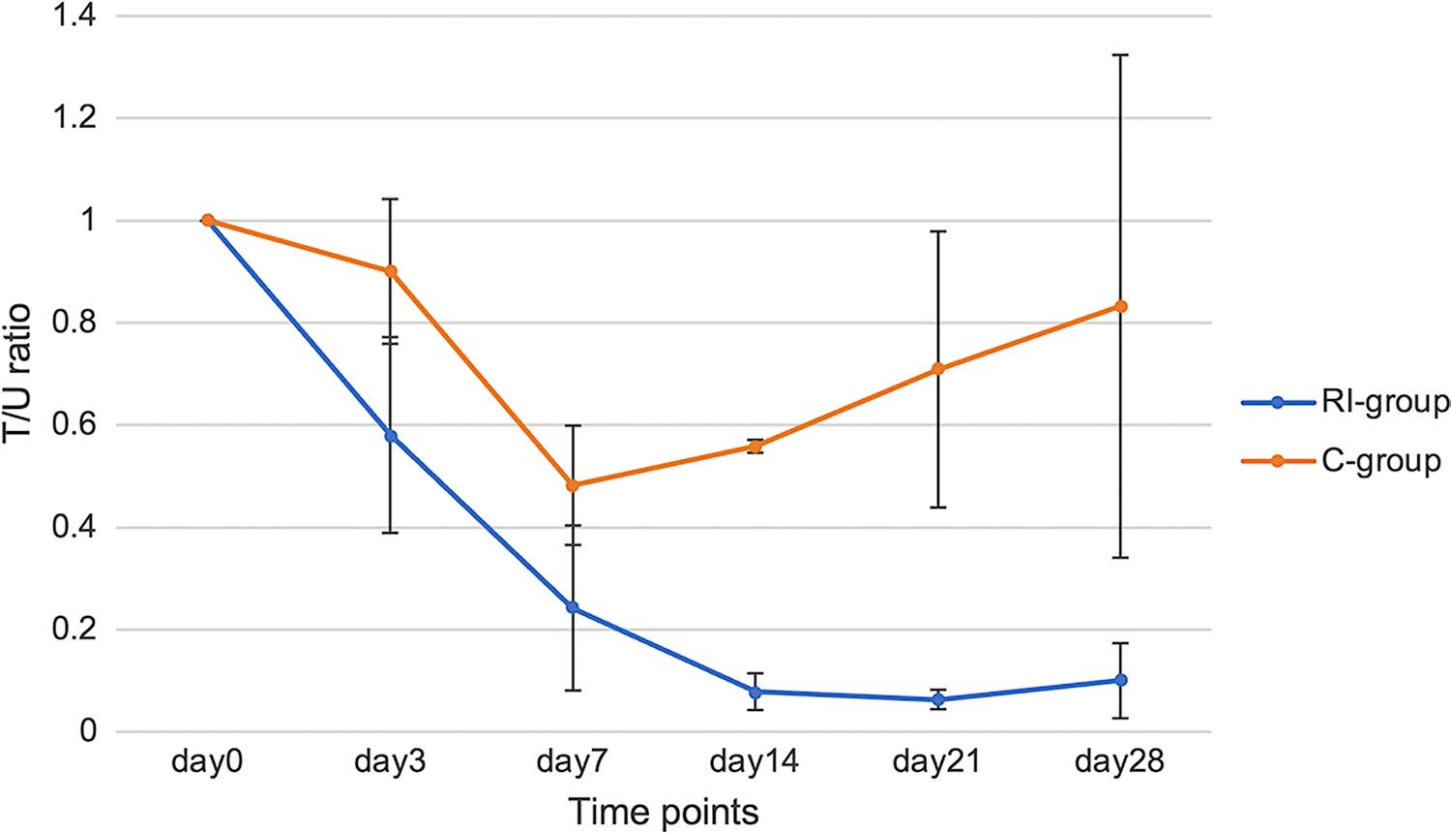
Treated-to-untreated tumor volume ratios (T/U) over time. RI-treated tumors exhibited significantly lower T/U ratios on days 14 (p = 0.017), 21 (p = 0.001), and 28 (p < 0.001), demonstrating sustained antitumor efficacy

The changes in the T/U ratio (Eq. (6)) over time are shown in Fig. 4. A reduction in tumor volume was observed on the treated side in both the RI-group and C-group when compared with the untreated side. However, a difference between the RI-group and C-group on the treated side was observed at days 14 and 28, and the T/U ratio was significantly higher in the RI-group (day14: p = 0.017, day 21: p = 0.001, day 29: p < 0.001).

The effect of time on the body weight change rate (Eq. (7)) was not statistically significant (p = 0.054), indicating no clear overall time-dependent difference in body weight change rate between the two groups. There was no significant difference in the weight change ratio at any specific time point relative to the baseline (day 0) in either group (Table 2). Additionally, there were no significant differences in weight change ratios between the RI-group and C-group at any time point. Post-treatment autopsy on day 28 revealed no visible abnormalities in the morphology or color of any organ.

**Table 2:** The body weight change ratios of the RI group and C group after treatment (unitless)

Time Point	RI-group	C-group	p-value	Adjusted p-value
day3	−0.067 ± 0.017	−0.077 ± 0.016	0.5389	1.000
day7	−0.053 ± 0.021	−0.089 ± 0.072	0.6108	1.000
day14	−0.028 ± 0.032	−0.068 ± 0.101	0.6723	1.000
day21	−0.028 ± 0.032	−0.036 ± 0.121	0.9407	1.000
day28	−0.022 ± 0.047	−0.042 ± 0.117	0.8529	1.000

Data are: Average ± standard deviation

## Discussion

This study investigated the therapeutic potential of ^177^Lu-lipiodol in HCC using a preclinical xenograft model and demonstrated a significant antitumor effect. The replication study revealed that gamma irradiation significantly reduced the proliferation rate of HC-4 cells, confirming their radiosensitivity. The reduction in replication was dose-dependent, with all irradiated groups showing marked suppression compared with the control group. This finding is consistent with previous studies indicating radiosensitivity of HCC [17, 18], thereby supporting the rationale for employing radionuclide therapy for HCC treatment.

### Stability and Biodistribution of ^177^Lu-Emulsified Lipiodol

The synthesis and emulsification of ^177^Lu-lipiodol yielded a high radiochemical purity (≥ 99.1%), ensuring stable tumor-specific delivery. SPECT/CT imaging revealed selective retention at the tumor site, with minimal off-target accumulation except for the spleen, which showed notably higher uptake and suggested a potential risk for hematologic toxicity. Although we observed no overt side effects in this pilot study, inadvertent local tissue damage or systemic toxicity could emerge if free ^177^Lu is released or if tumor margins are unclear. Therefore, future investigations should include blood count monitoring and organ function tests to fully characterize both the stability of ^177^Lu-lipiodol and its potential local and systemic toxicities.

### Tumor Growth Suppression and Antitumor Efficacy

Although ^177^Lu‐lipiodol was administered via direct femoral artery puncture under fluoroscopic guidance without selective catheterization of the tumor vessels for *in vivo* embolization, this study provided compelling evidence of the antitumor effects of ^177^Lu‐lipiodol. Nonetheless, our findings demonstrated significant tumor accumulation and therapeutic efficacy, despite the fact that this method did not allow selective catheterization of tumor‐feeding vessels (as in clinical TACE). Tumor size in the RI‐group exhibited significant reduction in growth compared with the untreated control group, particularly at day 21. Furthermore, T/U ratio analysis showed that the treated tumors maintained a reduction in growth until day 21, indicating the sustained therapeutic effect of ^177^Lu-lipiodol. On day 28, partial regrowth of the treated tumors was observed, which is consistent with the kinetics of radionuclide therapy. It is possible that multiple administrations rather than a single treatment may be required to achieve sustained tumor control.

### Comparison with Existing Modalities

^177^Lu‐lipiodol represents a promising alternative to other radiopharmaceutical lipiodol agents, such as iodine‐131 and rhenium‐188. Iodine‐131 has demonstrated efficacy in early studies, but its gamma emission properties are limited in therapeutic contexts, particularly in achieving sustained tumor targeting and patient convenience [19]. Rhenium‐188, with its higher beta energy (2.1 MeV) and shorter half‐life (≈17 h), has shown good liver retention and tumor selectivity in animal studies [20]. However, continuous radiation exposure to the tumor may not be adequately maintained due to its short therapeutic window [21]. In contrast, ^177^Lu combines optimal beta energy (0.497 MeV) with a moderate half‐life (≈6.7 d) and gamma emission suitable for SPECT imaging, which permits theragnostic monitoring of therapy. This advantage directly addresses a critical limitation of Yttrium-90 transarterial radioembolization (TARE), where real-time imaging is more difficult due to the lack of diagnostic gamma emissions. Although Yttrium-90 (max beta energy ~ 2.28 MeV) has been widely utilized in TARE, post-treatment verification of Yttrium-90 distribution often relies on bremsstrahlung imaging or PET-based detection of its positron fraction, complicating accurate dosimetry in real time [22]. Furthermore, the stability and high radiochemical yield of ^177^Lu‐lipiodol, as demonstrated in this study, support its potential for achieving prolonged tumor retention while minimizing systemic exposure. Dual therapeutic and imaging capabilities of ^177^Lu‐lipiodol suggest a distinct edge over both iodine‐131 and rhenium‐188, particularly in settings that require precise dosimetry and patient follow‐up. Thus, ^177^Lu‐lipiodol is a versatile and effective option for HCC treatment. Recently, alpha-emitting TARE approaches (e.g., Actinium-225-lipiodol) have shown potent tumoricidal effects due to their high linear energy transfer and short path length [23]. However, alpha therapies face challenges involving daughter-nuclide redistribution, supply-chain constraints, and potentially increased toxicity to the kidney. By contrast, ^177^Lu-lipiodol leverages a moderate beta range that can cover the oil-based embolic material’s distribution in the tumor, reducing the risk of missing residual tumor regions. Furthermore, ^177^Lu’s gamma co-emission and comparatively simpler handling enable safer monitoring, dose optimization, and potential multiple administrations—all critical when lipiodol rather than an internalizing antibody is used as the carrier. These features highlight the practical advantages of ^177^Lu-lipiodol in local tumor control with manageable systemic toxicity.

### Limitations and Future Directions

Despite these promising results, several limitations of this study remain to be addressed. First, the sample size was relatively small, which may limit the generalizability of the findings. Second, the observed tumor regrowth suggests that fractionated or repeated dosing regimens could further enhance therapeutic outcomes. Additionally, the high splenic accumulation observed in this study raises concern about possible hematologic toxicity, but hematologic parameters were not assessed in this pilot study. Future investigations should therefore include systematic blood examinations to clarify any potential toxicity. Finally, transitioning to clinical applications will require more extensive pharmacokinetic and dosimetric analyses, as well as larger-scale studies to thoroughly evaluate efficacy and safety.

## Conclusion

^177^Lu-lipiodol is a targeted radionuclide therapy for HCC demonstrating significant antitumor effects and favorable biodistribution.

## Supplementary Information

Below is the link to the electronic supplementary material.

Supplementary file1 (DOCX 16 KB)

## Data Availability

The datasets used and/or analyzed during the current study are available from the corresponding author on reasonable request.
