# Supplementary material for: Evaluation of 177Lu-Labeled Lipiodol as a Targeted Radionuclide Therapy for Hepatocellular Carcinoma in a Preclinical Xenograft Model
Source: Mol Imaging Biol. 2025 Jun 4;27(4):570–7. doi: 10.1007/s11307-025-02016-1 (PMC12405020; doi:10.1007/s11307-025-02016-1)
Supplement: Supplementary file 1 — Supplementary file1 (DOCX 16 KB) [file 11307_2025_2016_MOESM1_ESM.docx]

**Table 1: The tumor growth rate after treatment (unitless)**

| Time point | RI-group | C-group | p value | Adjusted p-value |
| --- | --- | --- | --- | --- |
| day0 | 1.0 ± 0.00 | 1.0 ± 0.00 |  |  |
| day3 | 0.85 ± 0.03 | 0.89 ± 0.00 | 0.789 | 1.000 |
| day7 | 0.56 ± 0.35 | 1.06 ± 0.24 | 0.210 | 1.000 |
| day14 | 0.45 ± 0.66 | 1.39 ± 0.71 | 0.060 | 0.360 |
| day21 | 0.59 ± 2.37 | 3.94 ± 3.06 | 0.002 | 0.012 |
| day28 | 1.60 ± 2.60 | 5.28 ± 3.30 | 0.022 | 0.133 |

Data are : Average ± standard deviation

**Table 2: The body weight change ratios of the RI group and C group after treatment (unitless)**

| Time Point | RI-group | C-group | p-value | Adjusted p-value |
| --- | --- | --- | --- | --- |
| day3 | -0.067 ± 0.017 | -0.077 ± 0.016 | 0.5389 | 1.000 |
| day7 | -0.053 ± 0.021 | -0.089 ± 0.072 | 0.6108 | 1.000 |
| day14 | -0.028 ± 0.032 | -0.068 ± 0.101 | 0.6723 | 1.000 |
| day21 | -0.028 ± 0.032 | -0.036 ± 0.121 | 0.9407 | 1.000 |
| day28 | -0.022 ± 0.047 | -0.042 ± 0.117 | 0.8529 | 1.000 |

Data are : Average ± standard deviation
